# Superconductor–insulator transition in capacitively coupled superconducting nanowires

**DOI:** 10.3762/bjnano.11.124

**Published:** 2020-09-14

**Authors:** Alex Latyshev, Andrew G Semenov, Andrei D Zaikin

**Affiliations:** 1 I.E. Tamm Department of Theoretical Physics, P.N. Lebedev Physical Institute, 119991 Moscow, Russia; 2 National Research University Higher School of Economics, 101000 Moscow, Russia; 3 Department of Physics, Moscow Pedagogical State University, 119435 Moscow, Russia; 4 Institute for Quantum Materials and Technologies, Karlsruhe Institute of Technology (KIT), 76021 Karlsruhe, Germany

**Keywords:** quantum phase slips, quantum phase transitions, RG equations

## Abstract

We investigate superconductor–insulator quantum phase transitions in ultrathin capacitively coupled superconducting nanowires with proliferating quantum phase slips. We derive a set of coupled Berezinskii–Kosterlitz–Thouless-like renormalization group equations demonstrating that interaction between quantum phase slips in one of the wires gets modified due to the effect of plasma modes propagating in another wire. As a result, the superconductor–insulator phase transition in each of the wires is controlled not only by its own parameters but also by those of the neighboring wire as well as by mutual capacitance. We argue that superconducting nanowires with properly chosen parameters may turn insulating once they are brought sufficiently close to each other.

## Introduction

Quantum fluctuations dominate the physics of superconducting nanowires at sufficiently low temperatures making their behavior markedly different from that of bulk superconductors [1–4]. Many interesting properties of such nanowires are attributed to the effect of quantum phase slips (QPSs) which correspond to fluctuation-induced local temporal suppression of the superconducting order parameter inside the wire accompanied by the phase slippage process and quantum fluctuations of the voltage in the form of pulses. By applying a bias current the symmetry between positive and negative voltage pulses is broken and, as a result, a superconducting nanowire acquires a non-vanishing electrical resistance down to the lowest temperatures [5–6]. This effect was directly observed in a number of experiments [7–10].

Likewise, quantum phase slips in superconducting nanowires yield shot noise of the voltage [11] which originates from the process of quantum tunneling of magnetic flux quanta across the wire. One can also proceed beyond the voltage–voltage correlator and evaluate all cumulants of the voltage operator, thus deriving full counting statistics of quantum phase slips [12]. This theory enables one to obtain a complete description of superconducting fluctuations in such nanowires. Interesting QPS-related effects also occur in superconducting nanorings which can be employed, for example, for possible realization of superconducting qubits [13]. Such effects were investigated theoretically [14] and observed experimentally [15–16].

Each quantum phase slip generates sound-like plasma modes [17] which propagate along the wire and interact with other QPSs. The exchange of such Mooij–Schön plasmons produces a logarithmic interaction in space–time between different QPSs where the magnitude is controlled by the wire diameter (cross section) [5]. For sufficiently thick wires this interaction is strong and the QPSs are bound in close pairs. Accordingly, the (linear) resistance of such wires tends to zero at *T* → 0, thus demonstrating a superconducting-like behavior in this limit. On the other hand, inter-QPS interaction in ultrathin wires is weak, quantum phase slips are unbound and the superconducting phase fluctuates strongly along the wire. In this case the wire looses long-scale superconducting properties, its total resistance remains non-zero and even tends to increase with decreasing temperature thus indicating an insulating behavior at *T* → 0. At zero temperature the transition between these two types of behavior comes as a quantum phase transition (QPT) driven by the wire diameter [5]. Below we will also refer to this QPT as a superconductor–insulator transition (SIT).

In this work we will show that this SIT can be substantially modified in a system of capacitively coupled superconducting nanowires even without any direct electrical contact between them. In our previous work [18] we already elucidated some non-local QPS-related effects in such nanowires which yield non-equilibrium voltage fluctuations in the system which exhibit a non-trivial dependence on frequency and bias current. Here we will demonstrate that quantum fluctuations in one of the two wires effectively ”add up” to those of another one, thereby shifting the QPT in each of the wires in a way to increase the parameter range for the insulating phase. Qualitatively the same effect is expected to occur in a single superconducting nanowire that has the form of a meander frequently used in experiments.

## Results and Discussion

### The model

**Figure 1:**
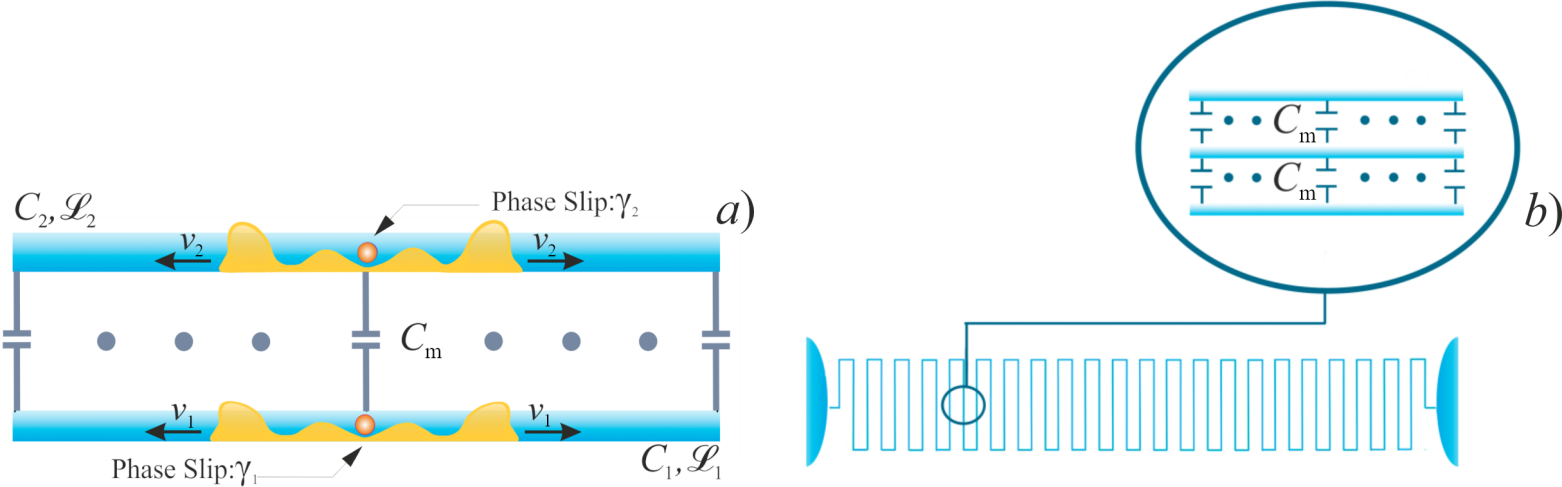
The systems under consideration: a) Two capacitively coupled superconducting nanowires and b) a superconducting nanowire in the form of a meander.

We first consider the system of two long superconducting nanowires parallel to each other, as schematically shown in Figure 1a.

The wires are described by geometric capacitances *C*_1_ and *C*_2_ (per unit wire length) and kinetic inductances 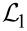 and 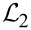 (times length) effectively representing the two transmission lines. Capacitive coupling between these two nanowires is accounted for by the mutual capacitance *C*_m_. The corresponding contribution to the system Hamiltonian that keeps track of both electric and magnetic energies in these coupled transmission lines reads

[1]
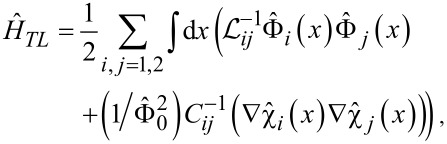


where *x* is the coordinate along the wires, 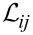 and *C_ij_* denote the matrix elements of the inductance and capacitance matrices

[2]
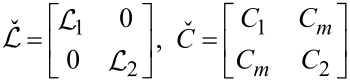


and Φ_0_ = π/*e* is the superconducting flux quantum. Note that for the sake of simplicity here and below we set Planck constant ℏ, speed of light, *c*, and Boltzmann constant, *k*_B_, equal to unity.

The Hamiltonian (Equation 1) is expressed in terms of the dual operators 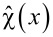 and 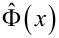
[14] which obey the canonical commutation relation

[3]
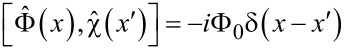


and are related to the charge density and the local phase operators, 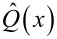 and 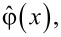 respectively, by means of the following equations

[4]
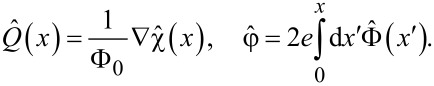


Physically, 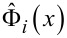 represents the magnetic flux operator, while the operator 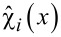 is proportional to that of the total charge 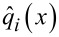 that has passed through the point *x* of the *i*th wire up to the time moment *t*, i.e., 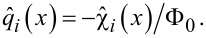


Provided that the wires are thick enough, the low energy Hamiltonian in Equation 1 is sufficient. However, for thinner wires, one should also account for the effect of quantum phase slips. The corresponding contribution to the total Hamiltonian for our system can be expressed in the form [14]

[5]
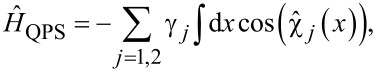


where

[6]
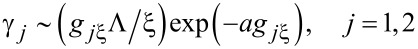


denotes the QPS amplitudes per unit wire length [6], *g_j_*_ξ_ = *R_q_*/*R_j_*_ξ_ is the dimensionless conductance of the *j*th wire segment of length equal to the superconducting coherence length ξ (here and below *R_q_* = 2π/*e*^2^ ≃ 25.8 kΩ is the quantum resistance unit and *R_j_*_ξ_ is the normal state resistance of the corresponding wire segment), Δ is the superconducting order parameter and *a* ≈ 1 is a numerical prefactor. We also note that the Hamiltonian (Equation 5) describes tunneling of the magnetic flux quantum, Φ_0_, across the wire and can be viewed as a linear combination of creation 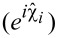 and annihilation 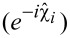 operators for the flux quantum Φ_0_.

It is obvious from Equation 4 that QPS events cause redistribution of charges inside the wire and generate pairs of voltage pulses moving simultaneously in the opposite direction (cf., Figure 1a)

[7]
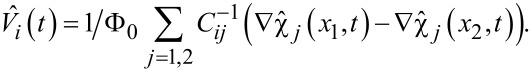


Clearly, in the presence of capacitive coupling quantum phase slips in one of the wires also generate voltage pulses in another one.

To summarize the above considerations, the total Hamiltonian for our system is defined as a sum of the two terms in Equation 1 and Equation 5,

[8]
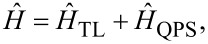


representing an effective sine-Gordon model that will be treated below.

### Quantum phase transitions: renormalization group analysis

In order to quantitatively describe QPT in coupled superconducting wires we will employ the renormalization group (RG) analysis. This approach is well developed and was successfully applied to a variety of problems in condensed matter theory, such as, the problem of weak Coulomb blockade in tunnel [19–22] and non-tunnel [23–25] barriers between normal metals or that of a dissipative phase transition in resistively shunted Josephson junctions [19,26–28]. In the case of superconducting nanowires QPT was described [5] with the aid of RG equations equivalent to those initially developed for two-dimensional superconducting films [29] which exhibit classical Berezinskii–Kosterlitz–Thouless (BKT) phase transition driven by temperature. In contrast, quantum SIT in quasi-one-dimensional superconducting wires [5] with geometric capacitance *C* and kinetic inductance 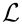 is controlled by the parameter [5]

[9]
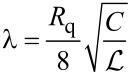


which is proportional to the square root of the wire cross section, *s*.

It follows immediately from the analysis of [5] that, provided the two superconducting wires depicted in Figure 1a are decoupled from each other (i.e., for *C*_m_ → 0), one should expect two independent QPTs to occur in these two wires respectively at λ_1_ = 2 and at λ_2_ = 2 where, according to Equation 9, we define 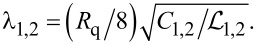 The task at hand is to investigate the effect of capacitive coupling between the wires on these two QPTs.

For this purpose let us express the grand partition function of our system 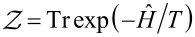 in terms of the path integral

[10]
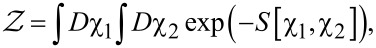


where

[11]
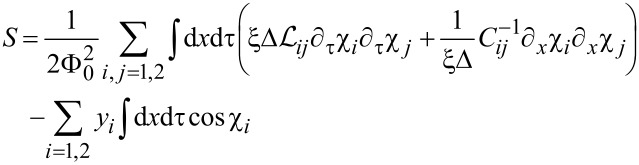


is the effective action corresponding to the Hamiltonian (Equation 8) and

[12]
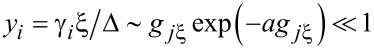


denotes the effective fugacity for the gas of quantum phase slips in the *i*th wire. Note that, having in mind that the QPS core size in *x*- and τ-directions is respectively *x*_0_ ∼ ξ and τ_0_ ∼ Δ^−1^, in Equation 11 for the sake of convenience we rescaled the spatial coordinate in units of *x*_0_, i.e. *x* → *x*ξ and the time coordinate in units of τ_0_, i.e. τ → τ/Δ.

In the spirit of Wilson’s RG approach we routinely divide the χ-variables into fast and slow components, 
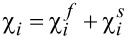
, where

[13]
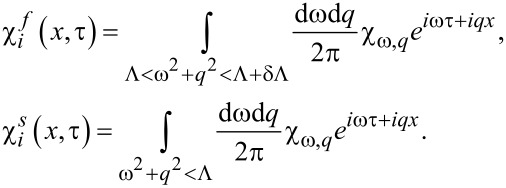


Setting δΛ/Λ ≪ 1, expanding in the fast field components 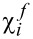 and integrating them out we proceed perturbatively in *y*_1,2_ and observe that in order to account for the leading order corrections it is necessary to evaluate the matrix Green function at coincident points which reads

[14]
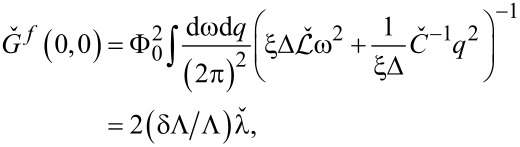


where 
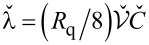
 and 
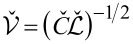
 is the velocity matrix for plasmon modes propagating along the wires. The matrix 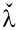 has the form

[15]
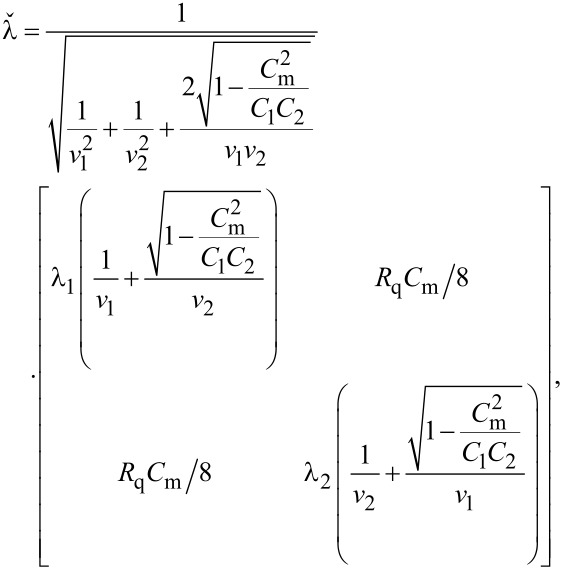


where 
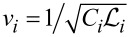
 is the velocity of the Mooij–Schön modes in the *i*th wire in the absence of capacitive coupling between the wires, i.e. for *C*_m_ → 0.

Following the standard procedure [29] and proceeding to bigger and bigger scales Λ, we eventually arrive at the following RG equations for the QPS fugacities *y*_1_ and *y*_2_:

[16]
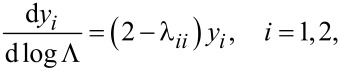


where λ_11_ and λ_22_ are diagonal elements of the matrix 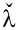 (Equation 15). Note that here we restrict our RG analysis to the lowest order in *y*_1,2_ which is sufficient for our purposes. As long as one keeps only the linear *y*_1,2_ terms in the RG equations, all other parameters of our problem, e.g., λ*_ii_*, remain un-renormalized.

As it can be observed from Equation 16, our system exhibits two BKT-like QPTs at λ_11_ = 2 and λ_22_ = 2. In the limit *C*_m_ → 0 the wires are independent from each other, λ_11(22)_ → λ_1(2)_ and these QPTs obviously reduce to that predicted in [5]. However, for non-zero capacitive coupling between the wires, the two QPTs occur at the values of λ_1,2_ exceeding 2. For the first wire the corresponding phase transition point is fixed by the condition

[17]
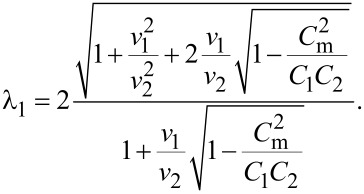


The same condition for the second wire is obtained from (Equation 17) by interchanging the indices 1 ↔ 2.

The above results allow us to conclude that in the presence of capacitive coupling SIT in both wires occurs at larger values of λ_1,2_ than in the absence of such coupling. In other words, quantum fluctuations in one of these wires effectively decrease the superconducting properties of the other one.

It follows from Equation 17 that the magnitude of such mutual influence depends on the ratio of the plasmon velocities in the two wires *v*_1_/*v*_2_ and on the strength of the capacitive coupling controlled by *C*_m_. Provided the wire cross sections *s*_1_ and *s*_2_ differ strongly the plasmon velocities 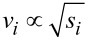 also differ considerably. Assume, for instance, that the first wire is much thinner than the second one. In this limit we have *v*_1_ ≪ *v*_2_ and, hence, the QPT condition (Equation 17) in the first wire remains almost unaffected for any capacitive coupling strength. If, on the contrary, the first wire is much thicker than the second one, then one has *v*_1_ ≫ *v*_2_ and the condition (Equation 17) reduces to 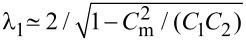 demonstrating that the critical value λ_1_ can exceed 2 considerably for sufficiently large *C*_m_ values.

**Figure 2:**
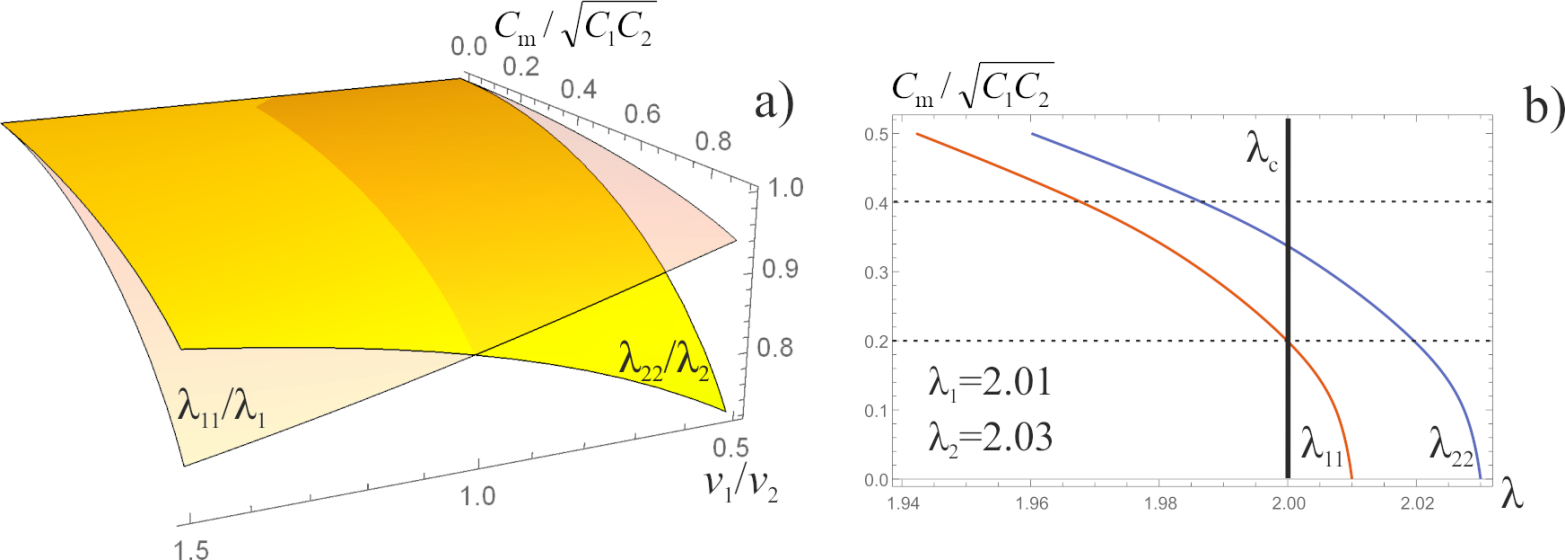
a) Critical surfaces corresponding to SIT at λ_11_ = 2 and λ_22_ = 2. b) Phase diagram for two capacitively coupled superconducting nanowires with λ_1_ = 2.01 and λ_2_ = 2.03. Both curves λ_11_(*C*_m_) and λ_22_(*C*_m_) decrease and cross the critical line λ_c_ = 2 with increasing mutual capacitance, *C*_m_.

It is obvious that the strength of capacitive coupling depends on the distance between the wires. At large distances this coupling is negligible *C*_m_ → 0. However, as the wires get closer to each other the value *C*_m_ increases and, hence, their mutual influence increases as well. Let us choose the wire parameters in such a way that for *C*_m_ = 0 both these wires remain in the superconducting phase being relatively close to SIT. In this case the parameters λ_1_ and λ_2_ should be just slightly larger than 2. Moving the wires closer to each other we ”turn on” the capacitive coupling between them, thus, decreasing both values λ_1_ and λ_2_ to less than 2. As a result, two superconducting wires become insulating as soon as they are brought sufficiently close to each other. This remarkable physical phenomenon is illustrated by the phase diagram in Figure 2b.

In order to complete this part of our analysis, we point out that transport properties can be investigated in exactly the same manner as was done in [5] in the case of a single nanowire. Generalization of the technique [5] to the case of two capacitively coupled superconducting nanowires is straightforward. For a linear resistance of the *i*th wire *R_i_*(*T*) and for λ*_ii_* *>* 2 (or for any λ*_ii_* at sufficiently high temperatures) we obtain

[18]
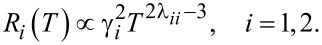


### Extension to other geometries

The effects discussed here can be observed in a variety of structures involving superconducting nanowires. For instance, superconducting nanowires in the form of a meander (see Figure 1b) are frequently employed in experiments [30]. In this case different segments of the wire are parallel to each other being close enough to develop electromagnetic coupling. Having in mind the above analysis, one expects that the wire of such a geometry would be ”less superconducting” than the same wire that has the form of a straight line.

For illustration, let us mimic the behavior of the wire depicted in Figure 1b by considering three identical capacitively coupled superconducting nanowires parallel to each other. For simplicity we will assume the nearest neighbor interaction, that is, the second (central) nanowire is coupled to both the first and the third nanowires via the mutual capacitance, *C*_m_, whereas the latter two are decoupled from each other. We again assume that the wires are thin enough and quantum phase slips may proliferate in each of these wires.

The quantum properties of this system are described by the same effective action (Equation 11) where the inductance and capacitance matrices now take the form

[19]
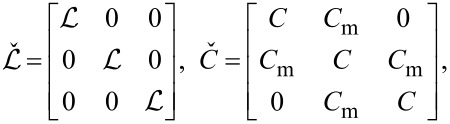


and the summation runs over the indices *i*,*j* = 1,2,3. Proceeding along the same lines as in the previous section we again arrive at Equation 14, where the diagonal elements of the matrix 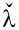 now read

[20]
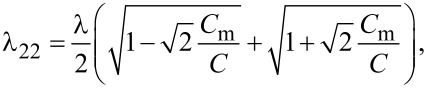


[21]
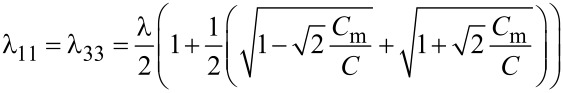


and the QPS interaction parameter λ is defined in Equation 9. We again arrive at the RG equations of the form (Equation 16) (now with *i* = 1,2,3). Being combined with Equation 20 and Equation 21 these RG equations demonstrate that in the presence of capacitive coupling SETs occur at λ*_ii_* = 2 implying λ *>* 2 for each of the three wires. This observation is fully consistent with our previous results derived for two coupled nanowires.

Furthermore, the RG equation (Equation 16) with *i* = 2 combined with Equation 20 also describes the effect of interacting quantum phase slips and QPTs in the wire having the form of a meander (Figure 1b). In this case, within the approximation of the nearest neighbor, capacitive interaction between the wire segments QPT occurs at

[22]
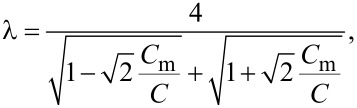


that is, the critical value of the parameter λ exceeds 2 as soon as the mutual capacitance *C*_m_ differs from zero. As it is clear from Equation 20 and Equation 21, the approximation of the nearest neighbor interaction appears to be well justified in the limit *C*_m_ ≪ *C*. For stronger interactions with *C*_m_ ≈ C this approximation most likely becomes insufficient for a quantitative analysis. However, on a qualitative level our key observations should hold also in this case: A nanowire in the form of a straight line with λ slightly exceeding the critical value 2 should demonstrate superconducting-like behavior with *R*(*T*) ∝ *T*^2λ−3^ [5] whereas a wire with exactly the same parameters may turn insulating provided it has the form of a meander with capacitive coupling between its segments.

## Conclusion

We have analyzed the effect of quantum fluctuations in capacitively coupled superconducting nanowires. We have demonstrated that plasma modes propagating in one such nanowire play the role of an effective quantum environment for another one, modifying the logarithmic interaction between quantum phase slips in this wire. As a result, the superconductor–insulator quantum phase transition gets shifted in a way to increase the parameter range for the insulating phase. Hence, superconducting nanowires may turn insulating provided they are brought close enough to each other. It would be interesting to observe this effect in forthcoming experiments with superconducting nanowires.
